# P-378. Trends in frequency of HIV viral load and CD4 cell count monitoring among Asian cohort of adults with HIV

**DOI:** 10.1093/ofid/ofaf695.596

**Published:** 2026-01-11

**Authors:** Mark Kristoffer U Pasayan, Awachana Jiamsakul, Evy Yunihastuti, Iskandar Azwa, Jun Yong Choi, Nagalingeswaran Kumarasamy, Sivaporn Gatechompol, Romanee Chaiwarith, Yu-Jiun Chan, Vohith Khol, Sasisopin Kiertiburanakul, Man-po Lee, I Ketut Agus Somia, Sanjay Putari, Cuong Duy Do, Thach Ngoc Pham, Suwimom Khusuwan, Oon Tek Ng, Junko Tanuma, Yasmin Gani, Rohidas Borse, Jeremy Ross, Rossana Ditangco

**Affiliations:** RESEARCH INSTITUTE FOR TROPICAL MEDICINE, CALAMBA, Laguna, Philippines; The Kirdy Institute, UNSW Sydney, Sydney, New South Wales, Australia; Faculty of Medicine Univesitas Indonesia, Jakarta, Jawa Barat, Indonesia; University of Malaya, Kuala Lumpur, Selangor, Malaysia; Yonsei University College of Medicine, Seoul, Seoul-t'ukpyolsi, Republic of Korea; VHS Infectious Diseases Medical Centre, Chennai Antiviral Research and Treatment Clinical Research Site, Chennai, India, Chennai, Tamil Nadu, India; Division of Infectious Diseases, Department of Medicine Faculty of Medicine, Ramathibodi Hospital, Mahidol University ., Bangkok, Krung Thep, Thailand; Faculty of Medicine, Chiang Mai University, Chiang Mai, Chiang Mai, Thailand; Taipei Veterans General Hospital, Taipei, Taipei, Taiwan; National Center for HIV/AIDS, Dermatology & STDs, Phnom Penh, Phnom Penh, Cambodia; Faculty of Medicine Ramathibodi Hospital, Mahidol University, Bangkok, Krung Thep, Thailand; Department of Medicine, Queen Elizabeth Hospital, Hong Kong SAR, China, Hong Kong SAR, China, Not Applicable, Hong Kong; Faculty of Medicine Udayana University & Sanglah Hospital, Bali, Bali, Indonesia; Institute of Infectious Diseases, Pune, Maharashtra, India; Bach Mai Hospital, Hanoi, Ha Noi, Vietnam; National Hospital for Tropical Diseases, HANOI, Ha Noi, Vietnam; Chiangrai Prachanukroh Hospital, Chiang Rai, Chiang Rai, Thailand; Tan Tock Seng Hospital, Singapore, Not Applicable, Singapore; National Center for Global Health and Medicine, Tokyo, Tokyo, Japan; Hospital Sungai Buloh, Sungai Buloh, Selangor, Malaysia; BJ Government Medical College and Sassoon General Hospital, Pune, Maharashtra, India; TREAT Asia, Bangkok, Krung Thep, Thailand; RESEARCH INSTITUTE FOR TROPICAL MEDICINE, CALAMBA, Laguna, Philippines

## Abstract

**Background:**

Viral load testing is recommended to monitor antiretroviral therapy effectiveness. This study examines changes overtime in the frequency of viral load and CD4 testing, as well as the relationship with AIDS diagnosis and mortality among an Asia-Pacific cohort of people with HIV.

**Methods:**

We included adults enrolled in the Treat Asia HIV Observational Database between 2003-2018 who were on ART for at least one year. VL and CD4 testing rates were analyzed using Poisson regression models. Association between testing frequency and AIDS diagnosis or survival were evaluated using Fine and Gray competing risk regression.

**Results:**

The analysis included 8446 patients. VL testing rates remained steady at 1 per person-year (PYS) between 2013-2018. Increased VL testing was associated with more frequent CD4 testing ( > 2 tests in the previous year; IRR=1.57, 95%CI 1.53-1.60), later follow-up years (2008-2012: IRR=1.15, 95%CI 1.12-1.18; 2013-2015: IRR=1.07, 95%CI 1.04-1.10), older age (31-40 years: IRR=1.06, 95%CI 1.03-1.08; 41-50 years: IRR=1.08, 95%CI 1.05-1.11; > 50 years: IRR=1.07, 95%CI 1.03-1.11), higher current VL (401-1000 copies/mL: IRR=1.16, 95%CI 1.09-1.24; > 1000 copies/mL: IRR=1.07, 95%CI 1.04-1.11), initial ART regimen (NRTI+PI: IRR=1.07, 95%CI 1.04-1.10; other combinations: IRR=1.11, 95%CI 1.05-1.17), and higher country income levels (upper-middle: IRR=2.17, 95%CI 2.11-2.23; high: IRR=3.14, 95%CI 3.03-3.26).

CD4 testing rates decreased from 2.04 to 1.06/PYS over the same period. Lower CD4 testing frequency was associated with HIV exposure mode (MSM: IRR=0.94, 95%CI 0.92-0.96; IDU: IRR=0.93, 95%CI 0.90-0.97; unknown: IRR=0.90, 95%CI 0.87-0.93), higher current CD4 (201-350 cells/µL: IRR=0.95, 95%CI 0.93-0.97; 351-500 cells/µL: IRR=0.89, 95%CI 0.87-0.91; > 500 cells/µL: IRR=0.85, 95%CI 0.83-0.87) and receiving an NRTI+PI first-line combination (IRR=0.96, 95% CI 0.94-0.98).

VL and CD4 testing frequencies were not significantly associated with AIDS diagnosis. However, having > 2 CD4 tests in the previous year was associated with higher mortality risks.

**Conclusion:**

Recognizing demographic, clinical and socio-economic factors affecting the frequency of CD4 and VL testing is critical to optimizing monitoring strategies and improving outcomes for PWH in the region.

**Disclosures:**

All Authors: No reported disclosures

